# Positron emission tomography in the detection of occult primary head and neck carcinoma: a retrospective study

**DOI:** 10.1186/1758-3284-4-34

**Published:** 2012-06-18

**Authors:** Gabriel Pereira, Joaquim Castro Silva, Eurico Monteiro

**Affiliations:** 1 Department of Otorhinolaryngology, Braga Hospital, Sete Fontes, 4710-243, Braga, Portugal; 2 Department of Otorhinolaryngology, Portuguese Institute of Oncology of Porto, Rua Dr António Bernardino de Almeida, 4200-072, Porto, Portugal

**Keywords:** Positron emission tomography, Unknown primary tumor, Head and neck carcinoma, Fluorodeoxyglucose, Metastases

## Abstract

**Background:**

The management of cervical lymph node metastases from an unknown primary tumor remains a controversial subject. Recently, Positron Emission Tomography (PET) has proved useful in the detection of these tumors, even after an unsuccessful conventional diagnostic workup. This study was performed to assess the role of PET in the detection of occult primary head and neck carcinomas.

**Methods:**

A retrospective analysis of a four year period at a tertiary referral oncology hospital was conducted.

**Results:**

Of the 49 patients with cervical metastases of carcinoma from an unknown primary, PET detected a primary in 9 patients and gave 5 false positive and 4 false negative results. Detection rate, sensitivity, specificity and accuracy were of 18.4%, 69.2%, 86.1% and 81.6%, respectively. PET was also of substantial benefit in detecting distant metastatic disease and, thus, altered therapeutic strategies in a significant amount of patients.

**Conclusions:**

Therefore, PET is a valuable tool in the management of patients with occult primary head and neck carcinoma, not only because it provides additional information as to the location of primary tumors, but also due to the fact that it can detect unexpected distant metastases.

## Background

Positron emission tomography (PET) is a functional image modality that characterizes the different tissues of the body according to perfusion and metabolic activity. 18 F-fluoro-2-deoxy-D-glucose (FDG), a radioactively labeled glucose analogue, is utilized due to its capacity to emit positrons that can be accurately localized by PET imaging. As tumor cells have an increased uptake of glucose, FDG accumulates within these cells, producing a “hot spot” on the PET image that can, therefore, be distinguished from surrounding normal tissue [1,2].

The utility of PET imaging has been demonstrated in the diagnosis and initial staging of head and neck tumors as well as in the evaluation of persistent or recurrent disease following radiotherapy [3,4]. Others have shown the benefit of PET in the detection of unknown primary head and neck cancer or synchronous primary tumors [5,6]. One advantage of PET over other imaging modalities, such as computed tomography (CT) or magnetic resonance imaging (MRI), is that, since PET imaging visualizes metabolic processes *in vivo*, relatively small tumors can be detected before structural changes have taken place, as long as they are metabolically active [7]. In fact, previously unapparent tumors, as small as 3 mm, have been detected by PET imaging [8]. PET can also differentiate normal from metastatic lymph nodes, sinus malignancy from secretions and tumor from fibrosis [7]. Furthermore, it is a non invasive technique that supplies full body information with only one session [9].

However, there are physiological areas of increased uptake in a normal PET scan which are prone to misinterpretation and can lead to false positive results. In the head and neck, these sites include the thyroid and salivary glands, muscles, Waldeyer’s ring and the brain [7]. False positive results can also be caused by inflammation [7]. Other disadvantages include the limited spatial resolution of PET scan which produces an anatomically inaccurate image. Significant improvement has been made at this level with PET/CT fusion technology, where PET imaging is supplemented by an overlay of a CT scan image, with improved sensibility and specificity [2,10].

Metastatic carcinoma in cervical lymph nodes of unknown primary origin is rare and accounts for only 3 to 5% of all head and neck tumors [2]. The most frequent histological finding is squamous cell carcinoma. Certain theories propose that there may not actually be a primary tumor in the aerodigestive tract and rather that the carcinoma has developed within a branchial cleft cyst or may have suffered spontaneous regression [11]. Although intriguing, little or no evidence exists to support these theories and it is more likely that there is, in fact, a subclinical primary tumor that cannot be detected by contemporary methods [2].

Traditional diagnostic evaluation for an unknown primary tumor consists of a thorough clinical examination including fiberoptic endoscopy of all the mucosa of the superior aerodigestive tract, CT and/or MRI followed by panendoscopy with directed biopsies and tonsillectomy [12]. Contrast-enhanced CT scans should cover the area from the skull base to the level of the thoracic inlet and either chest radiography or thoracic CT should be performed [2]. More recently, attention has been focused on PET using FDG in the diagnostic workup of these patients, although controversy still exists as to the real benefit [1,5,8,9,11,12]. This study was performed to clarify the potential role of PET in the detection of occult primary head and neck carcinoma.

## Methods

A retrospective study of all the patients diagnosed with an occult primary carcinoma of the head and neck region was conducted at the Francisco Gentil Portuguese Institute of Oncology of Porto (IPOPFG), a tertiary referral oncology hospital, within a 4 year period (between 2006 and 2009). All patients had histopathological proof of carcinoma of the cervical lymph nodes and had also undergone a comprehensive head and neck physical examination including fiberoptic endoscopy. Only patients, that had previously undergone CT evaluation of the head and neck and either chest radiography or thoracic CT to rule out a primary tumor, were eligible for this study. After the initial diagnostic evaluation had been negative for primary tumor, these patients had then undergone total-body PET imaging using FDG at the IPOPFG. Patients without confirmation of PET-positive findings (through tissue biopsies) were not included.

## Results

A total of 49 consecutive cases of occult primary head and neck carcinoma at the IPOPFG, between 2006 and 2009, met the above inclusion criteria. The medical records of these 49 patients were reviewed (Table 1). Forty-four (89.8%) were men and 5 (10.2%) were women. Overall, the mean age of the study group was 57.3 years, with a range between 36 and 81 years. The results of the neck staging included N1 in 8 patients, N2a in 10 patients, N2b in 10 patients, N2c in 1 patient and N3 in 20 patients. The histological diagnosis was of squamous cell carcinoma in 30 patients, poorly differentiated carcinoma in 12 patients, undifferentiated carcinoma in 3 patients and adenocarcinoma in 4 patients. With respect to topographical distribution, the upper and middle cervical lymph nodes were most frequently involved. Neck level II was involved in 75.5% of patients, whilst levels III and V were involved in 42.9% and 30.6% of cases, respectively. Levels IV and I were seldom found to harbor metastatic lymph nodes (16.3% and 10.2%, respectively) and no patient presented with involvement of level VI.

**Table 1:** Patient demographics.

**Patient no.**	**Sex**	**Age (yrs)**	**Tumor stage**	**Diagnosis (tumor type)**	**Localization of cervical lymph node metastases**	**PET result**	**Result of directed biopsy**	**Did PET result alter treatment strategy?**
1	M	55	N3	PDC	II, III	negative	n/a	No
2	M	57	N1	SCC	III	negative	n/a	No
3	M	65	N1	SCC	II	negative	n/a	No
4	M	65	N2a	SCC	II	negative	n/a	No
5	F	72	N2a	UC	II	negative	n/a	No
6	M	72	N3	SCC	V	pulmonary metastases	n/a	Yes
7	M	50	N1	SCC	II	hypopharynx	SCC	Yes
8	M	55	N3	SCC	II, III, V	negative	n/a	No
9	M	40	N1	SCC	II	oropharynx; bone and hepatic metastases	SCC	Yes
10	M	69	N2b	UC	II,III	bone and hepatic metastases	n/a	Yes
11	M	44	N3	SCC	II, III	negative	n/a	No
12	M	48	N2b	SCC	II	negative	n/a	No
13	M	70	N2a	SCC	II, III	negative	n/a	No
14	F	57	N1	PDC	I	base of tongue	SCC	Yes
15	M	48	N2a	PDC	II	nasopharynx; bone and extracervical lymph node metastases	negative	Yes
16	M	48	N3	Adenocarcinoma	II, III	negative	n/a	No
17	M	54	N3	SCC	II, III	negative	n/a	No
18	M	47	N2c	SCC	III, IV	hypopharynx	negative	No
19	M	57	N2b	PDC	II, III, V	hepatic metastases	n/a	Yes
20	M	68	N2a	SCC	III	supraglottis	negative	No
21	M	36	N3	Adenocarcinoma	II, III, IV, V	extracervical lymph node metastases	n/a	Yes
22	M	68	N3	SCC	II, V	negative	n/a	No
23	M	44	N3	SCC	II, III, IV, V	palatine tonsil	negative	No
24	M	45	N2a	PDC	II	extracervical lymph node metastases	n/a	Yes
25	F	56	N2b	Adenocarcinoma	V	bone and pulmonary metastases	n/a	Yes
26	M	53	N3	PDC	II, III	bone metastases	n/a	Yes
27	M	68	N3	SCC	II, III	lung (primary)	SCC	Yes
28	M	47	N2a	SCC	II	negative	n/a	No
29	M	46	N3	SCC	II, V	negative	n/a	No
30	M	75	N2b	PDC	I, II, V	parotid gland	SCC	Yes
31	M	55	N3	SCC	II, III	negative	n/a	No
32	M	56	N2b	PDC	II	negative	n/a	No
33	M	61	N2a	SCC	IV	negative	n/a	No
34	F	51	N3	SCC	II	palatine tonsil	SCC	Yes
35	M	48	N1	PDC	I	bone metastases	n/a	Yes
36	M	61	N3	SCC	II, III	negative	n/a	No
37	M	58	N2b	SCC	II, III	nasopharynx	negative	No
38	F	81	N1	SCC	IV	negative	n/a	No
39	M	78	N1	SCC	V	sinonasal primary and extracervical lymph node metastases	SCC	Yes
40	M	69	N2a	PDC	II	hepatic metastases	n/a	Yes
41	M	60	N3	SCC	II, III, IV	bone and extracervical lymph node metastases	n/a	No
42	M	52	N3	PDC	II, V	pulmonary and hepatic metastases	n/a	Yes
43	M	73	N3	SCC	II, V	negative	n/a	No
44	M	56	N3	PDC	II, III	bone and extracervical lymph node metastases	n/a	No
45	M	69	N3	SCC	IV, V	esophageal primary, bone and extracervical lymph node metastases	SCC	Yes
46	M	45	N2a	Adenocarcinoma	V	extracervical lymph node metastases	n/a	Yes
47	M	60	N2b	SCC	I, II	pulmonary, bone and extracervical lymph node metastases	n/a	No
48	M	54	N2b	SCC	I, II	base of tongue	SCC	Yes
49	M	44	N2b	UC	II, III, IV, V	negative	n/a	No

PDC: poorly differentiated carcinoma; SCC: squamous cell carcinoma; UC: undifferentiated carcinoma; n/a: not applicable.

**Figure 1:**
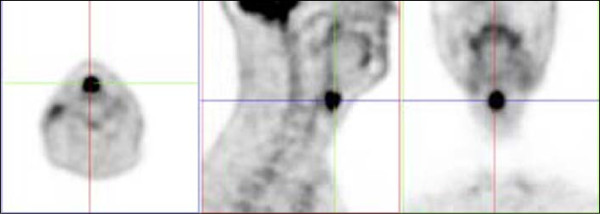
Case example of a false positive FDG-PET finding. Axial, sagittal and coronal views of the PET scan are displayed and demonstrate an uptake in the supraglottic region. The area was free of tumor when examined through careful panendoscopy and multiple deep directed biopsies were negative for neoplasia.

The PET scan was positive for cervical lymph node metastases in all 49 patients. In 14 patients, a possible primary tumor site was indicated by PET. Of these 14 patients, 9 were confirmed histopathologically through tissue biopsies as being squamous cell carcinomas. In the other 5 cases, directed biopsies were negative for tumor (false positive PET findings). Of the 9 primary tumors detected by PET, 4 were situated in the oropharynx (2 base of tongue, 1 palatine tonsil and 1 other oropharyngeal site), 1 in the hypopharynx, 1 in the sinonasal region, 1 in the parotid gland, 1 in the lung and 1 in the esophagus. Our overall detection rate was 18.4%. Of the 5 false positive results, 2 were located in the nasopharynx, 1 in the palatine tonsil, 1 in the hypopharynx and 1 in the supraglottis (Figure 1). Our overall false positive rate was 35.7%. On the other hand, PET detected possible distant metastases that had not been previously documented in 18 patients, which corresponds to a total of 36.7%. These sites included bone metastases in 10 patients, extracervical lymph nodes in 9, hepatic metastasis in 5 and pulmonary metastases in 4. Interestingly, 3 out of the 4 patients with adenocarcinoma had infraclavicular disease and patients with only lower neck involvement (areas IV or V) were also associated with a higher percentage of disease below the clavicles (47.4%).

In addition, 4 patients had false-negative PET findings with positive tissue biopsies. The mean follow-up time period surpassed between PET and primary tumor diagnosis was 10 months (with a range between 3 and 17 months). These were found to be squamous cell carcinomas of the palatine tonsil (2 cases) and piriform sinus (2 cases).

On a whole, the mean follow-up for all patients was 22.3 months. PET altered the treatment protocols in a total of 42.9% of patients. These changes, in 21 patients, were attributable to either the identification of a primary tumor, the detection of previously unknown metastases or both. Total PET results determined a sensitivity of 69.2%, a specificity of 86.1% and an accuracy of 81.6%.

## Discussion

Despite advanced radiological imaging methods, between 3 and 5% of all head and neck tumors will be diagnosed as being of unknown primary origin [2]. The vast majority are squamous cell carcinomas. Some authors have demonstrated that most of these tumors, when identified, are located in the palatine tonsil or the base of tongue area [13] and most advocate systematic bilateral tonsillectomy [14-16]. However, the real benefit of new imaging modalities and the validity of management strategies remain contreversial [2,11].

In order to reduce the heterogeneity of our study, we selected only those patients that had proven carcinoma in a cervical lymph node, excluding patients, for example, with melanomas and tumors of hematopoietic origin. With the intention of evaluating the additional benefit provided by PET over conventional radiologic imaging and workup, all patients included in this study had previously undergone complete clinical and endoscopic office examination, head and neck CT and, at least, chest radiography.

After conventional workup, our primary tumor detection rate with PET was found to be 18.4%. This corresponds to 9 patients with a histopathologically confirmed positive PET result out of a total 49 patients with unknown primary head and neck carcinoma. Detection rates vary in the literature from 5 to 73%, including a mean detection rate of 24.5% suggested by a large review [1]. Besides the ability to detect occult primary tumors, PET can also serve as a screening tool for distant synchronous primaries or metastatic disease [12]. Our PET results detected a primary squamous cell carcinoma of the lung and another in the esophagus. Although these could possibly be labeled as synchronous primaries with the true head and neck carcinoma remaining unknown, they were undetected by other imaging techniques and treatment strategies were significantly altered in these patients. Furthermore, possible distant metastases were identified in 18 patients (36.7%). This result is quite higher than that found in the literature [1,17], although this is probably due to the large number of advanced tumor stage (N3) cases in our series. The importance of whole body PET in the detection of distant disease should be stressed, especially when the lower neck is involved. In our study almost half (47.4%) of these patients had unexpected infraclavicular primaries or metastases and three of four patients with adenocarcinoma (75%) had pathology below the clavicles.

PET sensitivity in the current review (69.2%) was slightly lower than in most studies [1]. The 4 false negative results that were found emphasize the fact that a negative PET scan does not necessarily rule out the presence of a primary tumor and discard the need for further investigation [12]. Possible causes for such a low sensitivity in our review may be a low tumor uptake of FDG due to tumor differentiation or small size or a high background signal of Waldeyer’s ring [1]. This could, perhaps, justify the failure to detect the two cases of palatine tonsil carcinoma. However, there is always the possibility that there is no primary tumor to begin with, which would significantly reduce the number of true positives [9].

**Figure 2:**
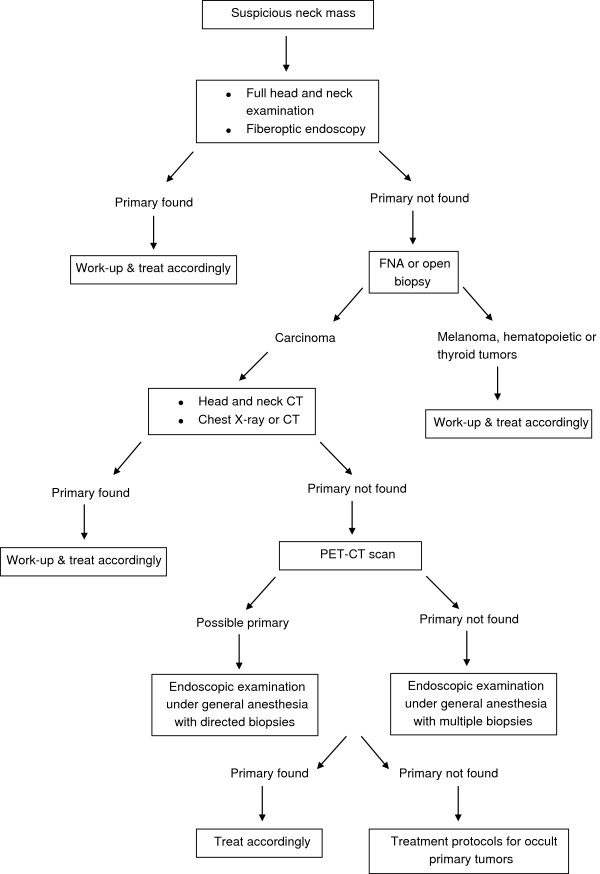
Management flow chart for unknown primary head and neck carcinoma.

One major weakness of PET in the detection of occult primary tumors is the high false positive rate and low specificity [1]. Due to the high percentage of false positives, some authors have found that there is a lack of benefit in using PET, but suggest that meticulous biopsy sampling and new tracers may ameliorate this aspect [11]. We obtained, in our series, a false positive rate of 35.7% and a specificity of 86.1%. This is consistent with or even somewhat better than previous reviews [1]. Nevertheless, the percentage of false positives is still fairly high. Proposed reasons include high physiologic uptake by the tonsils and muscles of mastication, inflammation and benign tumors [11,17]. Overall, PET helped identify 9 primary tumors which had previously gone undetected. It guided the surgeon to a potential primary tumor site for deep tissue biopsies. 31 patients were considered as having true negative PET scans, as no primary tumor was detected during a mean follow-up period of 22.3 months. This gives a total accuracy of 81.6%. A proposed management flow chart is shown in Figure 2.

Among other factors that must be considered when opting for PET imaging in the management of patients with unknown primary head and neck carcinoma are economic issues and availability. Most reports indicate that perhaps PET scanning is not cost effective [2]. Nonetheless, costs are comparable to whole body MRI [1]. On the other hand, no more than a few PET scans exist nationwide in Portugal, limiting availability to major referral centers.

## Conclusions

PET imaging is thus a valuable tool in the detection of occult primary head and neck carcinomas. Not only does it provide additional information as to the location of primary tumors, but it can also help detect unexpected distant metastases. As a result, therapeutic strategies and long-term prognosis are influenced by PET in a substantial number of these patients.

## Competing interests

The authors declare that they have no financial or non-financial competing interests.

## Authors’ contributions

The authors contributed equally to this study. GP drafted the manuscript. All authors critically reviewed, read and approved the final manuscript.

## Authors’ information

This study was carried out at the Portuguese Institute of Oncology of Porto.
